# Development and Preliminary Validation of the Parental Education in Physiotherapy Scale for Use in Spain: A Pilot Study

**DOI:** 10.3390/ejihpe16010005

**Published:** 2025-12-26

**Authors:** Manuel Pacheco-Molero, Irene León-Estrada, Catalina Morales-Murillo, Mónica Gutiérrez-Ortega

**Affiliations:** 1 Education and Psychobiology Department, Universidad Internacional de La Rioja, 26006 Logroño, Spain; manuel.pacheco@unir.net (M.P.-M.); irene.leon@unir.net (I.L.-E.); 2 INAT (Early Intervention Research Team—Equipo de Investigación en Atención Temprana), Universidad Internacional de La Rioja, 26006 Logroño, Spain; cp.morales@ucv.es; 3 Faculty of Education, Universidad Católica de Valencia San Vicente Mártir, 46001 València, Spain; 4 Pedagogy Department, Universidad de Valladolid, Paseo de Belén, 1, 47011 Valladolid, Spain

**Keywords:** psychometrics, factor analysis, physical therapists, paediatrics, patient education, self-assessment, patient-centred care

## Abstract

Given the scarcity of specific instruments to assess parental education in pediatric physiotherapy, this study developed the Parental Education in Physiotherapy Scale (Spanish acronym, EPF) and calculated its preliminary psychometric properties, acceptability, and feasibility. A cross-sectional instrumental study was conducted: the EPF was designed based on a specifications matrix and validated by expert judges in two phases. Then it was administered digitally to 63 physiotherapists experienced in treating children under six years of age. They rated it on a 4-point Likert response scale, and qualitative observations on its clarity, usefulness, and acceptability were collected. The validation showed adequate content validity (I-CVI ≥ 0.86; 31/32 items had I-CVI = 1.00) and high inter-judge agreement (global W = 0.659; *p* < 0.001). In the pilot study, exploratory factor analysis identified three factors (Collaboration, Capacity-Building, and Reflection) that explained 59% of the variance. After refining it, the final version comprised 18 items, showing high internal consistency (ordinal α = 0.944, ω = 0.934). Acceptability was high (100% found it useful; 98.4% said it facilitated reflection; 95.2% found it clear). Overall, the EPF provides preliminary evidence of validity, internal consistency, acceptability, and feasibility, although larger samples and additional analyses are required for its consolidation.

## 1. Introduction

Contemporary pediatric physiotherapy increasingly adopts family-centred intervention models, in which parents and other caregivers are recognised as active partners in the child’s development and are supported through capacity-building strategies (Rosenbaum, 2022). Recent systematic syntheses continue to support family-centred models in early intervention and pediatric rehabilitation, highlighting benefits for family engagement and caregiver empowerment (Jimenez-Arberas et al., 2024; Mestre et al., 2024; Mota et al., 2024). Sociocultural and social cognitive perspectives also support the family’s central role, as learning is mediated through interaction and scaffolding in everyday routines (Vygotsky, 1978) and facilitated by observational learning and caregiver self-efficacy (Bandura, 1986). As parental education in pediatric physiotherapy is enacted within daily routines and service systems, its implementation and impact are shaped by interacting influences at multiple levels (e.g., child characteristics, caregiver resources, professional practices, and organisational context). Accordingly, an ecological developmental framework can help to conceptualise these nested and reciprocal influences.

This perspective is underpinned by Bronfenbrenner’s ecological theory (Bronfenbrenner, 1979), which conceptualises development as shaped by dynamic interactions between the child and their wider family and contexts. This framework distinguishes the microsystem, mesosystem, exosystem, macrosystem, and chronosystem. The microsystem is particularly relevant here because parental education occurs within direct caregiver-child-professional interactions and everyday routines, where support can be most immediately implemented. Therefore, actions to enhance parental competencies have been shown to directly and positively impact the trajectory of child development, especially during the early years (Jackman et al., 2021).

However, the implementation of family-centred practices continues to be heterogeneous, with substantial variations between professionals and contexts of early care services (Adiguzel et al., 2022). Added to this are barriers such as families’ perception of insecurity and the limited availability of capacity-building resources (García-Ventura et al., 2023). In addition, to date, there are no specific validated instruments that evaluate the educational practice of pediatric physiotherapists with families. There are general questionnaires on family-centred practices, but these are mainly oriented toward families’ perceptions or satisfaction with the service, without directly addressing professionals’ educational behaviours or being psychometrically validated and adapted to this area (Peyton et al., 2022).

To address this need, the Parental Education in Physiotherapy (in Spanish, the “Escala de Educación Parental en Fisioterapia” [Spanish acronym, EPF]) scale was designed. This self-report instrument allows professionals to assess how often they inform, guide, and engage in family capacity-building with mothers, fathers, and other caregivers during interventions. From this approach, parental education is understood as the systematic process of transmitting knowledge, skills, and attitudes that enhance the families’ participation in their children’s functional development (Harniess et al., 2022).

Prior to its large-scale implementation, it is necessary to conduct a pilot study that explores the questionnaire’s feasibility and its initial psychometric properties, as recommended in the methodological guidelines for instrument development (Muñoz et al., 2024).

From this perspective, the findings from this exploratory phase will enable methodological optimization of the Parental Education in Physiotherapy scale, enhancing its theoretical, technical, and clinical relevance and contributing to the consolidation of family-centred interventions within pediatric physiotherapy (Medina-Valera et al., 2025).

It was hypothesised that the EPF would demonstrate adequate content validity, feasibility, and acceptability, and that its items would show a coherent multidimensional structure with good internal consistency in this pilot sample.

The objectives of this pilot study were: (1) to evaluate the content validity of the items of the EPF through multidisciplinary expert judgment, considering the criteria of clarity, relevance, and sufficiency (Mas et al., 2020); (2) to estimate the feasibility and acceptability of the self-administered digital format (McNamara et al., 2023); (3) to explore the emergent factor structure through exploratory factor analysis (EFA); and (4) to assess the internal consistency of the resulting dimensions.

## 2. Materials and Methods

### 2.1. Study Design

An instrumental pilot study was conducted using a quantitative, cross-sectional, exploratory design to develop and preliminarily validate the Parental Education in Physiotherapy (EPF) scale.

This cross-sectional instrumental pilot design (i.e., the design of the present study) is particularly appropriate in the early stages of instrument development, as it supports an initial empirical appraisal of item quality and the alignment between the proposed theoretical model and the observed data structure, in line with recommendations for preliminary psychometric validation (Streiner & Kottner, 2014).

In this study, there were three analytical blocks: (1) expert judgment for evidence of content validity and refinement; (2) acceptability/feasibility; and (3) EFA and reliability, for an initial structural approximation (DeVellis & Thorpe, 2021). This procedure of estimating reliability indices provides an initial insight into the internal consistency of instrument scores and guides decisions for subsequent confirmatory validation studies.

### 2.2. Participants

#### 2.2.1. Expert Judgment

Experts were recruited by purposive sampling based on their professional background and experience in early intervention and parental education. Panel size was defined according to content validity recommendations for the CVI, which suggest that panels of approximately 5–10 experts provide adequate agreement estimates (Lynn, 1986; Polit & Beck, 2006). To capture complementary perspectives, separate academic (*n* = 6) and clinical (*n* = 7) panels were used, both within the recommended range. Academic experts (Phase 1) and clinical pediatric physiotherapists (Phase 2) were invited via email using professional and institutional contacts, and eligibility was confirmed through a filter question on specific experience in parenting education.

The expert judgment was conducted in two complementary phases: an academic phase and a clinical phase, which were later followed by a field phase for pilot testing. The panel included professionals with backgrounds in physiotherapy, pedagogy, and psychology, all with extensive experience in attending to children aged 0–6 years. The status of “expert” was defined by a filter question on specific experience in parenting education. The composition and characteristics of both expert phases are presented in Table 1.

#### 2.2.2. Field Phase Participants

The sample comprised 63 licensed practicing pediatric physiotherapists in Spain, with a response rate of 78.7% (63/80), all of whom had clinical experience with children aged 0–6 years. Recruitment was not restricted to a single centre or institution and was conducted by self-selection; invitations were disseminated nationally through professional networks and associations using a survey link. Participation was voluntary and anonymous via Microsoft Forms after informed consent. Inclusion criteria were: (a) being a practicing physiotherapist; (b) having professional experience with the pediatric population aged 0–6 years; and (c) agreeing to participate by providing informed consent.

Mean age was 36.7 years (SD = 10.5; range 23–66), and mean professional experience was 12 years (SD = 9.36; range 0.5–38); 82.5% were women. In terms of academic training, 71.4% had postgraduate or doctoral studies, and 20.6% had received specific training in parental education (Table 2). There were no participant losses or incidents during data collection.

The sample size was established following the methodological recommendations for exploratory psychometric studies, which suggest a minimum of 5–10 participants per item or at least 50 cases as a threshold for a preliminary factor analysis (Everitt, 1975; Gorsuch, 2013). We acknowledge, however, that for the initial 32-item pool the present pilot sample (*n* = 63) yields a participant-to-item ratio of approximately 2:1, which is below common heuristics for stable factor solutions. Accordingly, in this pilot phase the EFA was used primarily as an initial item-reduction step, applying stringent retention criteria (primary loadings ≥ 0.60 and exclusion of problematic loading patterns), and the resulting factor structure is interpreted as preliminary. Replication in larger samples and confirmatory analyses are required to establish the stability of the dimensional solution (MacCallum et al., 1999; Mundfrom et al., 2005).

### 2.3. Instrument

The preliminary version of the Parental Education in Physiotherapy (EPF) scale was composed of 32 items, organised in a specification matrix that included eight indicators grouped into two initial theoretical dimensions: (a) Information and (b) Capacity-Building. Candidate items were generated through a structured instrument-development process. First, a conceptual review of the literature on family-centred practice and parental education in pediatric rehabilitation was undertaken to identify key professional behaviours expected in parental education delivered by pediatric physiotherapists (Harniess et al., 2022; Lillo-Navarro et al., 2015). Second, these behaviours were operationalised into a specification matrix comprising eight indicators distributed across the two dimensions. The Information dimension captured behaviours related to sharing, tailoring, and checking understanding of clinically meaningful information with parents/caregivers, whereas the Capacity-Building dimension captured behaviours focused on coaching, guided practice, problem-solving, and empowering families to implement strategies within everyday routines (Harniess et al., 2022; Lillo-Navarro et al., 2015). Third, items were drafted to describe observable behaviours in clinical practice and to avoid excessively abstract expressions, and then iteratively reviewed within the research team to ensure alignment with the theoretical definitions and to reduce redundancy.

A separate formal pretest (e.g., cognitive interviews) was not conducted; however, item clarity and content relevance were refined through the two-stage expert judgment process described in Section 2.4.1, and the final wording was further supported by participant feedback on clarity in the pilot acceptability assessment (Section 3.1).

The EPF uses a 4-point Likert-type response format with no neutral option (1 = almost never or never, 2 = occasionally, 3 = frequently, and 4 = almost always or always). This structure encourages a clear stance and reduces central tendency bias; evidence suggests that four to five response categories optimise measurement reliability/validity and discrimination among response options (Lozano et al., 2008). The scale was digitally administered, anonymous, and self-managed through Microsoft Forms, with an estimated completion time of 5–8 min. Prior evidence indicates that digital administration of Likert-type questionnaires is equivalent to paper formats in terms of reliability and validity (Gwaltney et al., 2008).

### 2.4. Procedure

This study included two sequential procedures: (1) expert judgment in two rounds for content validity; and (2) administration of the resulting version to participants (*n* = 63) to assess feasibility/acceptability, explore factor structure, and estimate internal consistency.

#### 2.4.1. Procedure 1. Expert Judgment

The process was carried out in two successive phases of expert judgment. In the first round of academic experts, the panel evaluated the theoretical and operational definitions and the consistency of the specification matrix. After the first round, drafting ambiguities were corrected. In the second phase, experts rated each item on a 4-point scale for each criterion (clarity, suitability, and relevance), where 1 = not clear/not suitable/not relevant, 2 = somewhat clear/suitable/relevant, 3 = clear/suitable/relevant, and 4 = very clear/highly suitable/highly relevant. For CVI computation, ratings of 3 or 4 were considered acceptable, following established content-validation recommendations (Lynn, 1986; Polit & Beck, 2006).

#### 2.4.2. Procedure 2. Administration to the Sample

The study was conducted in Spain using an online, self-administered survey. The sample was recruited through non-probabilistic sampling by self-selection, using a mixed dissemination strategy through professional networks, scientific associations, and digital platforms. The initial contact was by email, and participants accessed the questionnaire via Microsoft Forms after reading the information sheet and providing informed consent. This format was selected due to its accessibility, ease of use, capacity to ensure anonymous responses, and compatibility with data protection requirements. Data collection took place over a four-week period between November–December 2024. To maximise the response rate, two reminders were sent 15 days apart during the data-collection period.

In addition to the EPF scale, an ad hoc form with open questions was included to collect qualitative observations on item clarity, the instrument’s perceived usefulness, its possible negative risks, and acceptability for professional practice. These observations were analyzed through inductive thematic categorization, which allowed for identifying response patterns and guiding future drafting adjustments.

The study was approved by the relevant ethics committee [Universidad Internacional de La Rioja (PI: 066/2024)] and conducted in accordance with the principles of the Declaration of Helsinki and European Data Protection Regulations (GDPR).

### 2.5. Data Analysis

The statistical analysis was performed with the software from JASP 0.19.3 (JASP Team, 2024). An exploratory factor analysis (EFA) was applied to the polychoric correlation matrix, given the ordinal nature of the items of the Likert-type scale. The extraction method used was minimum residual with varimax rotation.

Minimum psychometric criteria were established: sample adequacy index KMO ≥ 0.60; significant Bartlett’s sphericity test (*p* < 0.001); factor loads ≥ 0.60 (Howard, 2016); and total explained variance greater than 50%. These thresholds were selected to ensure a level of demand consistent with exploratory psychometric studies in early stages (Hair et al., 2014). Content validity was quantified using the item-level content validity index (I-CVI). For each item and criterion, I-CVI was calculated as the proportion of experts who rated the item as 3 or 4 (acceptable) divided by the total number of experts (I-CVI = n [3–4]/N). Mean CVI values by dimension were computed as the average of the corresponding item-level CVIs. Kendall’s coefficient of concordance (W) was used as a complementary index of inter-judge agreement.

In addition, the descriptive statistics of the items (frequencies, means, standard deviation, skewness, and kurtosis) were calculated to identify ceiling or floor effects. Likewise, reliability indices were estimated for the scale and its factors: ordinal α and McDonald’s ω, because the former is sensitive to the number of items and the tau-equivalence assumption, whereas the latter offers a more robust estimate of internal consistency (Dunn et al., 2014). Floor and ceiling effects were examined by calculating, for each item, the percentage of responses in the lowest (1) and highest (4) categories. A floor or ceiling effect was considered potentially relevant if more than 15% of responses were concentrated at the extreme.

The qualitative observations collected through the ad hoc form were analyzed with an inductive thematic categorization approach, which identified recurring patterns in the participants’ appraisals of the instrument’s clarity, usefulness, and acceptability.

## 3. Results

### 3.1. Content Validity

The quantitative analysis yielded a content validity index (CVI) ≥ 0.86 in all items, exceeding the minimum recommended threshold (0.80); 31 out of the 32 items reached a maximum score (CVI = 1.00) (Table 3). Kendall’s coefficient of concordance (W) showed a high degree of agreement among experts (global W = 0.659), with particularly high values in the criteria of suitability (W = 0.978) and relevance (W = 0.999).

The results showed mean content validity indices (CVI) ≥ 0.80 in the two dimensions of Information and Capacity-Building (Table 3), exceeding the recommended threshold of 0.78 for panels of ≥6 experts (Polit & Beck, 2006). Given that the clinical expert panel comprised seven members, an I-CVI of 1.00 indicates unanimous endorsement (7/7 experts rating 3 or 4). We acknowledge that the combination of a small panel and dichotomization may contribute to high I-CVI values; therefore, these results are interpreted as supportive but preliminary evidence of content validity. In this case, the reported CVIs correspond to the second phase of the trial (clinical panel, *n* = 7). The inter-judge agreement, estimated by Kendall’s W, was statistically significant (*p* < 0.001), which supports the homogeneity of the assessments. Thus, the CVI values were adequate, and the inter-judge agreement was significant. Following the experts’ recommendations, semantic adjustments were made to 21 items to enhance the linguistic clarity and conceptual soundness of the instrument, resulting in the initial operational version used in this pilot phase.

### 3.2. Feasibility and Acceptability

The scale showed high acceptability among the participants (*n* = 63). Specifically, 100% considered it useful, 98.4% indicated that it facilitated reflection, 98.4% rated the completion time as adequate, and 95.2% perceived clarity in the behaviours.

Of the qualitative comments, 11 were obtained through inductive thematic categorization, which reinforced the positive assessment linked to acceptability and identified areas for improvement linked to feasibility: (a) need for personalization (e.g., including other caregivers in the drafting of the items) and (b) greater clarity in some items (the items referred to were not specified in the commentary).

No evidence of social desirability bias or effects attributable to the Likert format was observed, either in the questionnaire responses or in the qualitative feedback (including the specific question about negative effects). Taken together, these results support the acceptability and feasibility of the scale for application in clinical and research contexts, in accordance with methodological recommendations for pilot phases of validation of psychometric instruments (Boateng et al., 2018).

### 3.3. Factor Structure and Internal Consistency

The emerging factors were labelled through a content-based interpretation of the retained items with the highest loadings. Factor 1 was named Collaboration because its items reflect a partnership-oriented process consistent with family-centred pediatric rehabilitation, where families and professionals engage in bidirectional exchange of knowledge, shared responsibility, and shared decision-making around goals and intervention. Factor 2 was named Capacity-Building because its items represent participatory/coaching practices designed to strengthen caregivers’ competence and confidence to implement strategies within everyday routines. Factor 3 was named Reflection because its items capture reflective appraisal and planning (e.g., inviting families to evaluate actions, identify adjustments, and anticipate next steps), aligning with established models of reflective practice in professional education.

The KMO index was 0.64, a value considered acceptable, at the lower limit recommended for this type of analysis. Bartlett’s sphericity test was significant (χ^2^ = 9354.91; df = 496; *p* < 0.001), supporting the relevance of the EFA (Nkansah, 2018). Items with factor loads < 0.60 or with cross-loadings were removed, resulting in an intermediate 18-item version (Table 4).

For transparency, the following 14 items from the initial 32-item pool were removed at this stage because they did not meet the predefined EFA criteria (i.e., primary loading < 0.60 and/or a problematic loading pattern in this pilot sample): I3, I4, I5, I6, I7, I12, I15, I16, C6, C7, C8, C9, C11, and C13. The remaining 18 items met the conservative retention threshold and were retained for the refined version (Table 4).

The three factors jointly explained 59% of the total variance (Table 5). Collaboration accounted for 21% of the variance and comprised items reflecting partnership-oriented behaviours with families (e.g., bidirectional exchange and shared discussion). Capacity-Building accounted for 17% of the variance and included items describing coaching-oriented practices aimed at strengthening families’ competence to implement strategies in everyday routines. Reflection accounted for 21% of the variance and comprised items focused on reviewing actions with families and planning next steps between sessions. In addition to the structural analysis, the internal consistency of the 18-item refined version was estimated. The overall reliability of the scale was high: ordinal α = 0.944 and ω = 0.934. By factors, α ranged between 0.825 and 0.881, and ω between 0.870 and 0.906, indicating adequate reliability (George & Mallery, 2019). Regarding score distributions, floor and ceiling effects were examined for the retained items by estimating the proportion of responses in the minimum (1) and maximum (4) categories. The observed distributions did not indicate problematic clustering at the extremes according to the predefined criterion, supporting adequate variability for preliminary interpretation in this pilot sample.

## 4. Discussion

Family-centred pediatric physiotherapy still lacks specific instruments to assess how parental education behaviours are incorporated in clinical practice (Peyton et al., 2022; Ribeiro & Snider, 2022). This pilot study provides preliminary evidence on the internal structure, reliability, acceptability, and feasibility of the Parental Education in Physiotherapy (EPF) scale, which enables pediatric physiotherapists to self-assess educational and coaching practices with caregivers within family-centred services.

The findings can be situated within the broader literature assessing family-centred service delivery in pediatric rehabilitation. For example, the Measure of Processes of Care (MPOC) and its service-provider version (MPOC-SP) have been used to capture family-centred behaviours and to support professional development and programme evaluation (Cunningham & Rosenbaum, 2014). However, these instruments are not specific to parental education behaviours within pediatric physiotherapy. The EPF was designed to address this gap by focusing specifically on the educational and coaching practices that physiotherapists use to support parents/caregivers as active agents of change in the child’s everyday contexts.

Recent implementation work also indicates that limited shared knowledge and inconsistent application remain barriers to family-centred care in pediatric rehabilitation settings, reinforcing the need for practical, profession-specific tools to support reflection and training (Nematifard et al., 2024). In the Spanish early intervention context, families have also reported the relevance of family-centred practices and natural environments, underscoring the value of tools that help professionals operationalise these principles in routine practice (Montaño-Merchán et al., 2025).

Content review by two complementary expert panels supported the relevance and clarity of the initial item pool and led to targeted wording refinements, strengthening interpretability for pediatric physiotherapy contexts. The refined EPF domains can be interpreted as reflecting three core components of family-centred parental education: (i) collaboration, capturing partnership processes such as reciprocal information exchange and shared decision-making; (ii) capacity-building, reflecting coaching-oriented support that facilitates caregiver implementation within daily routines; and (iii) reflection, focusing on guided appraisal and planning to promote learning across sessions. This pattern is consistent with conceptual accounts of family-centred service delivery and reflective practice (Schön, 1983; Mann et al., 2009) and complements broader measures such as the MPOC/MPOC-SP by focusing specifically on parental education behaviours within pediatric physiotherapy. Given the pilot nature of the study, the dimensional structure should be interpreted cautiously and confirmed in larger samples using confirmatory analyses.

Reflection captures review and prospective planning with families regarding actions between sessions. This domain aligns with established reflective practice models (Schön, 1983) and with syntheses in health professions education that emphasise reflection as a mechanism supporting learning and practice improvement (Mann et al., 2009). Within the EPF, this domain reflects intentional facilitation of caregiver appraisal, adjustment, and planning, which is congruent with coaching-oriented practice.

The exploratory factor analysis supported refinement of the initial 32-item, two-domain version (Information and Capacity-Building) into an 18-item version organised into three domains: Collaboration, Capacity-Building, and Reflection. The emergence of three domains may reflect how parental education is enacted in family-centred physiotherapy. Practices initially conceptualised as “information” provision are often embedded in reciprocal, relationship-based exchanges (e.g., negotiation of priorities, shared understanding, and shared decision-making) and may therefore cluster with collaboration rather than forming a distinct dimension. In addition, items referring to guided appraisal and planning may capture a reflective facilitation process aligned with coaching-oriented practice, which can differentiate from both information exchange and capacity-building. In early-stage instrument development, initial conceptual models are also commonly refined when empirical analyses and item reduction identify more differentiated practice domains; accordingly, the proposed structure should be confirmed in larger samples using confirmatory approaches.

This three-factor structure appears conceptually coherent within this pilot sample; however, given the preliminary nature of the study and the sample size, it should be interpreted cautiously and requires replication with larger, more diverse samples and subsequent confirmatory analyses to establish the stability of the factor solution. The labelling of the three factors follows established conceptualisations in family-centred practice and professional education. Evidence from this pilot sample supports an interpretable three-domain solution; however, the limited sample size indicates that the structure should be confirmed in larger and more diverse samples using confirmatory approaches (Nkansah, 2018).

Purpose and clinical application. The EPF is intended as a practical self-assessment tool for pediatric physiotherapists working within family-centred services. It can be used in early intervention, outpatient and community pediatric rehabilitation services, hospital-based pediatric physiotherapy, and private practice settings. Potential objectives include guiding reflective practice and clinical supervision, identifying professional development needs across collaboration, capacity-building, and reflective facilitation, monitoring change following training initiatives, and supporting service evaluation and research on implementation of family-centred parental education practices. By providing a structured profile across the three domains, the EPF may help clinicians identify strengths and prioritise specific, actionable targets for improving how they support families within everyday routines.

Recommendations. The EPF can be used in routine practice as a structured prompt for self-reflection and supervision, helping clinicians identify strengths and specific targets across collaboration, capacity-building, and reflective facilitation. In service settings, aggregated EPF profiles may inform team training priorities and support the evaluation of professional development initiatives aimed at strengthening caregiver participation in everyday routines. Recent evidence supports the use of parent coaching and telecoaching formats to facilitate caregiver implementation and engagement, suggesting that EPF-guided training and feedback could be integrated within these delivery models (Chien et al., 2024; Hurtubise et al., 2025). For education and training, the three domains provide an organising framework to design competency-based modules (e.g., shared decision-making and partnership communication; coaching strategies for routine-based implementation; and guided reflection and action planning with families). For research, the next steps include confirming the three-domain structure in larger and more diverse samples using confirmatory approaches, testing stability over time (test–retest), and examining convergent and discriminant validity with related measures of family-centred practice. Future implementation studies should also explore responsiveness to training and whether EPF-guided feedback is associated with improvements in caregiver engagement and routine-based practice.

The qualitative analysis of the observations collected identified the necessary terminological adjustments and editorial improvements that were not detectable in the quantitative analyses. This process supported the instrument’s acceptability and feasibility in real contexts, aligning with emerging practices that integrate quantitative and qualitative evidence when developing instruments focused on user experience (Boateng et al., 2018).

Overall, the pilot phase suggests that the EPF can provide a structured way to characterise and reflect on parental education practices in pediatric physiotherapy, complementing existing family-centred measures by targeting education and coaching behaviours. This focus may support professional self-reflection, supervision, and training initiatives aimed at strengthening caregiver participation within everyday routines.

Future research should confirm the factor structure with larger, more representative samples and incorporate additional sources of validity evidence, including confirmatory construct validation, convergent and discriminant validity, criterion-related validity where appropriate, and test–retest reliability, to consolidate the EPF as a robust tool that can be transferred to different clinical and academic contexts.

### Limitations

A first limitation of the study was the use of non-probabilistic sampling by self-selection, which could introduce participation biases by favoring the inclusion of physiotherapists with greater interest or familiarity with parental education. This may have reduced the variability of the responses and limited the representativeness of the sample. In addition, no information was collected on the geographical distribution by autonomous community or on the work setting (public/private), which limits the sample’s nature and the generalization of the results.

However, although the sample size (*n* = 63) was sufficient to perform a preliminary EFA, the participant-to-item ratio for the initial pool was low (≈2:1), which may limit the stability and generalisability of the extracted factor structure. Therefore, these findings should be replicated in larger samples and tested using confirmatory approaches before the dimensional structure is considered definitive.

Additionally, this pilot phase did not examine several recommended sources of validity evidence, including confirmatory construct validation (e.g., CFA), convergent/discriminant validity, criterion validity, or formal item discrimination analyses. These will be addressed in subsequent phases with larger and more diverse samples. Test–retest reliability was not assessed in this study because the survey was administered anonymously and no identifiers were collected to link responses across time, which prevented re-contact and longitudinal matching; test–retest assessment is planned for a future phase using a re-contactable sampling strategy.

## 5. Conclusions

The Parental Education in Physiotherapy (EPF) scale showed preliminary evidence of content validity, coherent factor structure at the theoretical and practical levels, high internal reliability, and adequate acceptability and feasibility in its digital application. These results support its potential as a professional self-assessment and research tool in pediatric physiotherapy, contributing to the visibility and systematization of the assessment of family-centred educational practices in this field.

Given the pilot nature of the study and the methodological limitations identified, the findings should be interpreted with caution. Future phases should replicate the analysis in larger and more diverse samples, apply confirmatory factor analysis, and explore evidence of convergent and discriminant validity to consolidate the EPF as a robust psychometric scale.

In short, the EPF constitutes a relevant initial step towards the systematic integration of parental education in pediatric physiotherapy, with direct implications for clinical practice, professional capacity building, and research aimed at family-centred approaches.

## Figures and Tables

**Table 1:** Characteristics of the participants in the expert judgment.

	Phase 1 (Academic)	Phase 2 (Clinical)
Nr. of Participants		
Women	5	7
Men	1	-
Age		
Age range	28–66	31–66
Mean	46	47
Academic training		
Diploma/Degree	-	2
Postgraduate	-	2
PhD	6	3
Degree		
Physiotherapy	4	7
Pedagogy	1	-
Psychology	1	-
Years of experience with children aged 0–6 years (mean)	16	23
Experts in parental education	4 (66%)	7 (100%)
Target population ^1^	-	5

^1^ Target population: pediatric physical therapists who perform their current clinical practice with families.

**Table 2:** Sociodemographic and professional characteristics of participants (*n* = 63).

	*n*	(%)	Mean	SD ^1^
Sex				
Woman	52	(82.5%)		
Man	11	(17.4%)		
Academic training				
Diploma	8	(12.6%)		
Degree	10	(15.8%)		
Postgraduate	42	(66.7%)		
PhD	3	(04.7%)		
Parenting education training				
Yes	13	(20.6%)		
No	50	(79.4%)		
Age (years)	63		36.7	10.5
Professional experience (years)	63		12.0	9.36

^1^ SD = standard deviation.

**Table 3:** Content validity index of the initial version of the Parental Education in Physiotherapy Scale, composed of 32 items, after the second expert judgment.

Domain	Items	Content Validity Index (CVI)					
		Clarity		Suitability		Relevance	
		CVI	Nr. Rating 3 or 4	CVI	Nr. Rating 3 or 4	CVI	Nr. Rating 3 or 4
Information	1	1.00	7	1.00	7	1.00	7
	2	1.00	7	1.00	7	1.00	7
	3	1.00	7	1.00	7	1.00	7
	4	0.86	6	1.00	7	1.00	7
	5	1.00	7	1.00	7	1.00	7
	6	1.00	7	1.00	7	1.00	7
	7	1.00	7	1.00	7	1.00	7
	8	1.00	7	1.00	7	1.00	7
	9	1.00	7	1.00	7	1.00	7
	10	1.00	7	1.00	7	1.00	7
	11	1.00	7	1.00	7	1.00	7
	12	1.00	7	1.00	7	1.00	7
	13	1.00	7	1.00	7	1.00	7
	14	1.00	7	1.00	7	1.00	7
	15	1.00	7	1.00	7	1.00	7
	16	1.00	7	1.00	7	1.00	7
Capacity-Building	17	1.00	7	1.00	7	1.00	7
	18	1.00	7	1.00	7	1.00	7
	19	1.00	7	1.00	7	1.00	7
	20	1.00	7	1.00	7	1.00	7
	21	1.00	7	1.00	7	1.00	7
	22	1.00	7	1.00	7	1.00	7
	23	1.00	7	1.00	7	1.00	7
	24	1.00	7	1.00	7	1.00	7
	25	1.00	7	1.00	7	1.00	7
	26	1.00	7	1.00	7	1.00	7
	27	1.00	7	1.00	7	1.00	7
	28	1.00	7	1.00	7	1.00	7
	29	1.00	7	1.00	7	1.00	7
	30	1.00	7	1.00	7	1.00	7
	31	1.00	7	1.00	7	1.00	7
	32	1.00	7	1.00	7	1.00	7

**Table 4:** Rotated solution of the exploratory factor analysis of the items of the initial version of the EPF scale.

Item	Item Description	Factor 1	Factor 2	Factor 3	Uniqueness
I1	I inform the family of the child’s functioning during the joint evaluation.	0.176	0.493	**0.667**	0.281
I2	I describe to the family the strengths, interests, and preferences I observe in the child in the joint assessment.	0.148	**0.608**	0.438	0.417
I3	I provide the family with written information about what was observed in the child in the joint evaluation.	0.174	0.354	0.065	0.840
I4	I inform the family, according to scientific evidence and clinical practice guidelines, about the current situation and prognosis of the child’s diagnosed health condition.	0.254	0.543	0.227	0.589
I5	I present options to the family for actions and available resources to address the child’s health condition and motor performance.	0.184	0.591	0.439	0.424
I6	I describe the advantages and disadvantages, according to scientific evidence, of the actions and available resources.	0.141	0.427	0.320	0.695
I7	I tailor the information according to the ability and preferences of the child and family.	−0.159	0.181	0.529	0.662
I8	I explain to the family in detail what the actions and available resources consist of.	0.119	0.413	**0.630**	0.418
I9	I explain the necessary involvement of the family in the proposed actions.	**0.629**	0.269	0.576	0.200
I10	I highlight the key aspects of the actions to be carried out by the family in the child’s normal setting.	**0.694**	0.417	0.459	0.133
I11	I coordinate with the family to determine the place and time of day when the proposed actions are required.	**0.628**	0.449	0.332	0.294
I12	I explain the benefits that can be obtained from the proposed actions.	0.568	0.122	0.525	0.387
I13	In workshops or meetings with families, I promote the exchange of relevant information related to health and challenges in children’s motor development.	**0.911**	0.128	0.105	0.143
I14	In the workshops or meetings with families, I encourage the exchange of experiences on the health condition and the challenges in children’s motor development.	**0.921**	0.052	0.026	0.148
I15	I share with groups of families, according to scientific evidence, knowledge on the application of functional, preventive and/or therapeutic interventions, as well as the use of support products.	0.551	0.244	0.050	0.635
I16	I guide families about the available health, educational, and/or community resources and programs.	0.470	0.336	0.251	0.603
C1	I promote the family’s observation during the demonstration with the child of the actions to be performed by the family in the child’s normal setting.	0.126	0.298	**0.680**	0.433
C2	I invite the family to practice the actions previously explained and demonstrated in the session.	0.459	0.157	**0.658**	0.331
C3	I indicate to the family the actions that have been performed in the session.	0.434	0.142	**0.671**	0.341
C4	I encourage the family to make adjustments to the actions performed in the session.	0.603	0.180	**0.637**	0.198
C5	I invite the family to comment on what they have observed from the actions they have performed in the session.	0.333	**0.629**	0.399	0.335
C6	I use audiovisual material (from the family itself or designed material) so that the family will comment on the actions performed in the session.	0.091	0.495	0.203	0.706
C7	I encourage the family to identify the necessary adjustments to be made in the child’s actions or normal setting.	0.379	0.585	0.346	0.395
C8	I encourage the family to comment on the actions performed in the session.	0.477	0.451	0.454	0.363
C9	I resolve the doubts raised by the family about the actions to be performed in the child’s normal setting.	0.383	0.247	0.399	0.634
C10	I ask the family to indicate what actions they are going to practice in their usual setting until the next session.	0.574	**0.696**	0.138	0.167
C11	I ask the family to describe the actions to be performed in the child’s normal setting until the next session.	0.552	0.595	0.160	0.315
C12	I ask the family to describe the key components of the actions to be performed.	0.416	**0.821**	0.126	0.136
C13	I talk with the family about how these actions can benefit their child’s motor development and health.	0.128	0.574	0.264	0.585
C14	I appraise with the family how these actions can improve their child’s quality of life and participation in daily activities.	−0.003	**0.617**	0.351	0.496
C15	I discuss with the family how we will evaluate the possible changes that occur in their child.	0.118	**0.724**	0.049	0.460
C16	I ask what results they hope to obtain at the family level, not only for the child and the actions to be performed.	**0.684**	0.258	0.076	0.460

Note: The retained items (loads ≥ 0.60) are indicated in bold; the rest were discarded. C = Capacity-Building; I = Information.

**Table 5:** Factor structure of the EPF (18 items), internal consistency (α, ω), and explained variance.

Factor	Number of Items	Example of Representative Items	α	ω	Explained Variance (%)
Collaboration	6	I promote the exchange of relevant information related to health and challenges in children’s motor development.	0.881	0.906	21
Capacity-Building	6	I invite the family to practice the actions previously explained and demonstrated in the session.	0.825	0.882	17
Reflection	6	I ask the family to indicate what actions they are going to practice in their usual setting until the next session.	0.839	0.870	21
Total	18		0.944	0.934	59

Note: α = ordinal alpha; ω = McDonald’s omega.

## Data Availability

The data presented in this study are available on request from the corresponding author.

## Abbreviations

The following abbreviations are used in this manuscript:

EPF	Parental Education in Physiotherapy Scale (Spanish acronym)
EFA	Exploratory Factor Analysis
CVI	Content Validity Index
